# Influence of Quackery on Health

**Published:** 1856-06

**Authors:** 


### INFLUENCE OF QUACKERY ON HEALTH.

WE take the following pungent extract from a speech by Mr. Sanborn, in the New-Hampshire Legislature, upon the bill to incorporate the "New-Hampshire Medical Botanical Society:"

"It is safe to assert that there is not an advertised nostrum in the market which does not hold out false hopes to the sick. Every such advertisement is an imposition upon the public, whether it came from physicians regular, irregular, or defective, and in the grammar of medicine the latter class is very numerous. If one tithe of what the patent medicine makers assert were true, we might attain unto what the progenitors of our race would have secured by partaking of the fruit of the tree of life. We might live forever if the pompous assertions of the makers of cosmetics, washes for the face and beautifying lotions were true, we might have ladies as beautiful as houris, with the assurance of perpetual juvenescence. In a word, we might bid defiance to the darts of death, and the vegetable doctor might stand over the prostrate king of terrors and exclaim in triumph, 'O death, where is thy sting?' and then turn to his patient and in the language of Oriental adulation exclaim, 'O patient, live forever.'

"It is pretended that nobody is deceived by the professions of quacks. Every day's experience contradicts this assertion. The rich and the poor, the wise and the simple, are all occasionally deluded by these cheating, lying impostors. The human mind is so constituted that we must confide in others. We are made to trust each other, to believe the solemn declarations of our fellows. Without this mutual cnnfidence, society could not exist; hence the abuse of it becomes the more odious. None are so credulous as the sick. They listen readiy to the advice and suggestions of others. Fearing the ravages of disease, they eagerly lay hold of any hope, however delusive, which empirics may hold out to them. The extensive sale of vegetable medicines proves this. A few years ago, when Morrison's vegetable life pills were so popular in this country, a suit was commenced in a court in Massachusetts, by Morrison & Moat, against John K. Palmer, for selling a spurious article. It appeared there in evidence that the proprietors had been so successful in England as to be able to establish the 'British College of Health,' at an expense of $250,000, from which agents were sent into all the principal cities of Europe and America. The demand for these pills became so great in this country that the sale amounted to $250,000 in a single year; and the seller of spurious pills had disposed of one hundred thousand boxes before he was arrested by the patentee. It appeared, furthermore, that this 'British College of Health,' with its high sounding title, had neither charter, professors nor students, but consisted of an immense building in the suburbs of London, with appropriate apparatus for the manufacture of 'Hygean pills;" and that the proprietor was neither surgeon, physician nor man of science, but an arch quack. What has become of his vaunted remedy in the brief space of ten years? Gone, like thousands of its predecessors, to the shades of Erebus and old Night.

The fact that new nostrums remain popular only for a brief period proves that their healing virtues, like the diseases they profess to cure, are *imaginary*. Each remedy has its brief day of glory, and is succeeded by a rival candidate for the popular applause. Each new invention has a twofold office. It comes to bury the dead and herald a new race. Every fresh adventurer denounces all rivals as deceivers and imposters. These makers and venders of nostrums abuse each other like pickpockets. They wage upon every fellow-quack an internecine war. Every member of the fraternity is an Ishmælite to every other. On all sides it is war to the knife, and knife to the hilt. The dead lie prostrate on many a hard fought field? but it is the *patients* who die, not the *quacks!* But are we not bound to believe what these impostors say of each other? Who should know the tricks of the trade better than they? if we can trust their promises we certainly are bound to credit their assertions concerning the fraternity. They warn us "*as we value health*," to shun all prescriptions of quacks except their own; and this is done by every inventor of a new medicine. Look at the flaming advertisements of the rival Drs. Townsend, which stare us in the face from every paper printed in Concord, together with a beautiful wood-cut, representing old Dr. Jacob Townsend himself. They both offer for sale a syrup of sarsaparilla. The old Doctor says he has paid $200,000 within the last eight years for advertising; and whence came this immense sum? We cannot suppose that any man would devote more than a tithe of his income to advertising: therefore, the doctor must have been doing an excellent business in the sarsaparilla line for eight years.

At the present day there is a great fondness for vegetabe, medicines. Any thing having the prefix of vegetable to it goes down with the multitude. Notwithstanding every body knows that no new vegetable has been discovered, and no new properties have been detected in vegetables before known, still they confide in the assertion that the commonest herbs may be made soverelgn remedies for "all the ills that flesh is heir to." It is equally well known that a majority of all the medicines in the pharmacopœia of the regular faculty are of vegetable origin, and that the most deadly poisons, such as destroy life almost at a blow, like a thunder-bolt, are from the vegetable kingdom.—Still we are told that all vegetable remedies are safe, while mercury is the great bugbear of the many. But it has been proved in courts of justice, where quacks have been arraigned for manslaughter, that pills professing to be purely vegetable have produced *salvation* in the patient. There are perhaps, a score of infallible remedies for consumption, and there can scarcely be a doubt that the only ingredient in them all which serves to allay the irritation of a chronic cough is *opium!* This for a time quiets the consumptive patient, and deceives him with the hope of recovery, but by frequent use of it the strength is exhausted, and the system sinks under the repeated assaults of empiricism.

But, of all the gross and palpable impositions upon the public credulity, the pretence that the Indians understood the healing virtues of roots and herbs, is the most absurd and monstrous. Civilized and Christian men having recourse to savages to learn science! It is, however, a notorious fact that Indian " medicine men, as they are called, are the greatest impostors living. They surpass their civilized imitators. They " out-herod Herod," in knavery. The whole system of practice ameng the Indians has always consisted in fraud and pretense. Catlin, who spent years among the North American Indians, constantly affirms this. They know literally nothing of the power of simples. They employ, over the sick, charms, spells and incantations, and make use of amulets and consecrated medicine bags as curative agents. Yet our scientific botanists go to these ignorant, besotted dupes of superstition to learn medical science. Sometimes a veritable Indian doctor appears among us with more *brass* than *copper* in his face. He makes his prescription with great gravity and solemnity. He cuts his herbs and gathers his roots under the influence of certain astronomical signs. These signs by the way, are but a relic of old astrology, as ancient as the Pharaohs, and have no more significancy for us than the worship of Isis. But our doctor regards " the stellar" influence in gathering his herbs. He strips the bark *upward* for an emetic, and *downward* for a cathartic. He steeps the whole in river water taken up in a peculiar way. I once heard of an instance where the whole process failed because the patient dipped the water up stream instead of down! " Because you see," said the learned doctor, " if the water be dipped up stream it goes *agin* natur; if down stream it *helps* natur." Such are Indian doctors. *Ab uno disce omnes.*

